# Epistasis and evolution: recent advances and an outlook for prediction

**DOI:** 10.1186/s12915-023-01585-3

**Published:** 2023-05-24

**Authors:** Milo S. Johnson, Gautam Reddy, Michael M. Desai

**Affiliations:** 1 Department of Integrative Biology, University of California, Berkeley, CA USA; 2 Biological Systems and Engineering Division, Lawrence Berkeley National Laboratory, Berkeley, CA USA; 3 Physics & Informatics Laboratories, NTT Research, Inc., Sunnyvale, CA USA; 4 Center for Brain Science, Harvard University, Cambridge, MA USA; 5 Department of Organismic and Evolutionary Biology and Department of Physics, Harvard University, Cambridge, MA USA

**Keywords:** Macroscopic epistasis, Microscopic epistasis, Adaptability, Robustness, Evolvability

## Abstract

As organisms evolve, the effects of mutations change as a result of epistatic interactions with other mutations accumulated along the line of descent. This can lead to shifts in adaptability or robustness that ultimately shape subsequent evolution. Here, we review recent advances in measuring, modeling, and predicting epistasis along evolutionary trajectories, both in microbial cells and single proteins. We focus on simple patterns of global epistasis that emerge in this data, in which the effects of mutations can be predicted by a small number of variables. The emergence of these patterns offers promise for efforts to model epistasis and predict evolution.

## Predicting evolution requires an understanding of epistasis

**Fig. 1:**
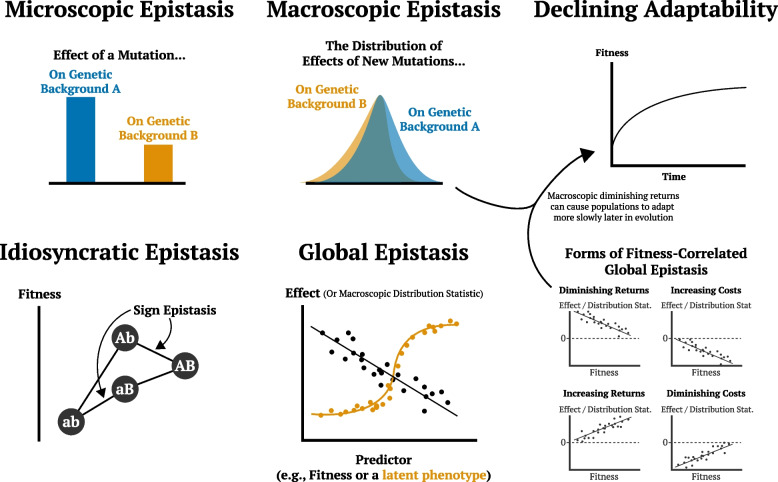
Illustration of types of epistasis. See Box 1 for definitions of these terms

Individual mutations often have phenotypic effects that vary widely depending on the genetic background in which they occur, due to interactions with other variants both within the same gene and elsewhere in the genome (Fig. 1; Box 1). These genetic interactions, known as epistasis, can provide clues into functional relationships and physical interactions within and between important proteins, pathways, and modules within the cell. For example, epistatic interactions can reflect physical contacts between residues within protein complexes or alterations in the function of enzymes involved in coupled metabolic pathways. This has driven widespread interest in systematic screens for epistasis (e.g., between all pairs of gene deletions in yeast [1]). Numerous studies have shown that epistatic interactions are also common among mutations that accumulate along the line of descent in adapting laboratory microbial populations [2, 3], and play an important role in a variety of natural evolutionary processes (e.g., in maintaining binding to host cell receptors during the evolution of antibody escape in SARS-CoV-2 [4-6]). Epistasis has the potential to shape the course of evolution by opening up or closing off potential adaptive trajectories, and by collectively shifting the entire distribution of fitness effects (DFE) of new mutations available to the adapting population.

**Fig. 2:**
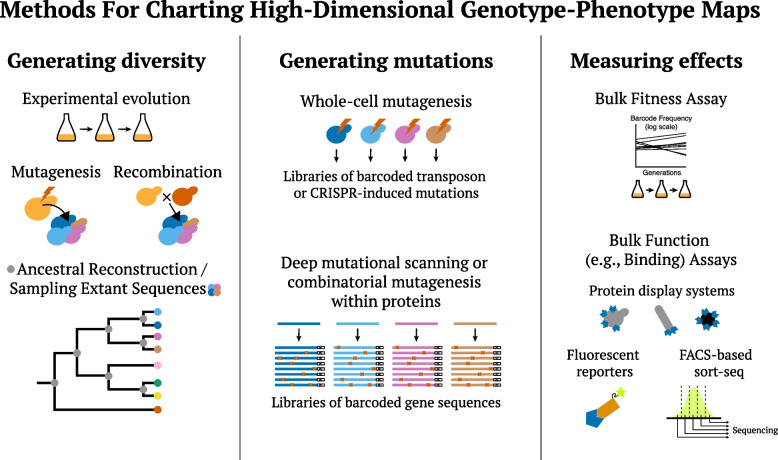
Illustration of methods for measuring the fitness effects of a large number of mutations in a number of genetic backgrounds

The DFE influences the dynamics of adaptation — for example, the rate of fitness increase or the accumulation of deleterious load — in analytically tractable ways [7-9]. Thus predicting evolutionary dynamics requires predicting how this distribution changes as mutations accumulate during evolution. In recent years, high-throughput experimental techniques have begun to make it possible to empirically quantify these effects (Fig. 2). This body of work suggests that, even when individual epistatic interactions between specific mutations are largely unpredictable, we may still be able to predict “macroscopic” changes in the DFE as populations evolve, at least in the context of relatively short-term evolution of laboratory microbial populations [10-13]. However, it remains unclear how broadly these patterns will apply across other biological systems or over longer evolutionary timescales.

Whether we are trying to predict how the DFE changes during short-term laboratory adaptation or how the effect of one mutation will differ in two distantly related strain backgrounds, we face a difficult, high-dimensional problem. In the worst-case scenario, the fitness effect of each mutation depends irreducibly on the state of every other locus in the genome. In a best-case scenario, mutations combine additively with respect to fitness, such that predicting the fitness effect of a mutation is as simple as measuring its effect once on any genetic background. Recent evidence suggests that the reality lies between these two extremes: epistasis is common, but structured in a way that sometimes allows us to predict the effects of mutations from relatively few predictors or parameters. These simplified forms of epistasis have been generically termed “global,” “nonspecific,” or “unidimensional” [14-16], though the specific phenomenology depends on the context.

In microbial evolution, the single best predictor of mutational effects is often fitness itself: the effects of mutations and certain aspects of the DFE can sometimes be predicted based only on the fitness of the genetic background on which these effects are measured. The most commonly observed form of fitness-correlated epistasis in microbes is “diminishing-returns,” in which a beneficial mutation is less beneficial on more fit backgrounds (Box 1).

In this review, we start with recent studies describing examples of diminishing-returns epistasis, before turning to consider how this work motivates broader questions about epistasis and evolution:

1. How often do we see fitness-correlated epistasis during evolution? What other patterns of global epistasis emerge over shorter and longer evolutionary timescales?
2. What models can explain global epistasis?
3. Can we infer patterns of global epistasis and use them to predict phenotypes or forecast evolutionary outcomes?

## Patterns of global epistasis in microbial evolution experiments

### Diminishing-returns epistasis can explain declining adaptability in evolving populations

A common observation in experimental microbial evolution is declining adaptability: populations tend to adapt quicker at the beginning of an evolution experiment. Further, among closely related strains, the best predictor of adaptability is often the initial fitness of the population, such that populations which have lower initial fitness adapt more quickly (reviewed in Couce and Tenaillon [17]). This pattern is best reflected in the *E. coli* long-term evolution experiment (LTEE), where the rate of fitness increase has declined dramatically and reproducibly across tens of thousands of generations [10, 18]. Declining adaptability has also been observed in yeast strains that differ by a few mutations [14], tens of thousands of mutations [19], in different bacteriophage isolates [20] and in panels of bacteria, phages, and yeast that have disruptive or deleterious mutations (often referred to as compensatory adaptation or repair experiments [21-27]).

A potential explanation for patterns of declining adaptability is diminishing-returns epistasis. Diminishing-returns epistasis often manifests as a simple negative linear relationship between the fitness effect of individual mutations and the background fitness and has been observed in many microbial evolution experiments [2, 3, 13, 14, 27-34]. Wünsche et al. [12] showed that the average strength of beneficial mutations sharply decreased as populations gained fitness in the LTEE by using marker divergence experiments to track the prevalence and effects of beneficial mutations. This experiment convincingly showed that declining adaptability results *primarily* from a shift in the beneficial DFE due to epistasis, in contrast to the scenario where the number of beneficial mutations decreases while their effects remain constant. Several recent transposon mutagenesis studies have shown a reduction in the beneficial DFE during the LTEE, though we should note that these studies only include loss-of-function mutations [35-37]. A recent study in yeast has also shown that fixation of even a single beneficial mutation can lead to a shift towards a DFE with less beneficial mutations [13].

While evidence for declining adaptability and diminishing-returns epistasis is now widespread, it is useful to identify exceptions to this rule. For example, when Rojas Echenique et al. [24] allowed yeast gene-deletion strains to evolve, initial fitness predicted some, but not all, of the variation in the rate of adaptation: this study found that the functional module in which genes were deleted also played a role (i.e., in some cases two strains with similar initial fitness adapted at significantly different rates). Smith et al. [38] evolved four highly diverged *E. coli* strains (the two most diverged strains only shared 57% of their genes), starting populations with one of three engineered mutations, and found that while declining adaptability held across mutated populations of each strain (when initial genotypes differed by single mutations), it did not hold across strains. However, we note that in Kavvas et al. [39] the initially less fit genotypes among a different panel of *E. coli* strains did gain more fitness during evolution than their higher-initial-fitness counterparts. In contrast to diminishing-returns epistasis, many studies have reported specific cases of synergistic epistasis between beneficial mutations that drive important evolutionary dynamics [3, 40-43]. Thus a key question, which we will return to later in this review, is when and why we should expect to find patterns of diminishing-returns epistasis, and when we might expect to see something different.

### Increasing-costs epistasis can lead to a reduction in mutational robustness during evolution

We recently used a transposon mutagenesis system in yeast to show that a set of 91 mostly deleterious insertion mutations became, on average, more deleterious over the course of 10,000 generations of evolution in one lab environment [11]. We had previously used this library to show that these mutations also tend to be more deleterious in higher-fitness strains isolated from a cross between two diverged yeast strains [44]. In both cases, these DFE-level patterns did not emerge from a consistent decrease in the fitness effects of each mutation. Instead, we observed highly variable patterns of epistasis: some mutations have a constant effect independent of genetic background (no epistasis), some have specific interactions with individual loci, and others show consistent patterns of diminishing-returns, increasing-costs, or decreasing-costs epistasis. While increasing-costs epistasis was widespread enough to cause consistent changes to the DFE in the strains isolated from the yeast cross and from one of our evolution environments, fewer mutations showed this trend in another evolution environment, suggesting that the nature of selection acting on the evolving population can influence whether this trend is reliably observed.

Three recent studies have used transposon mutagenesis to study how the fitness effects of mutations change during the LTEE, focusing on overall changes to the distribution of fitness effects [35], changes in gene essentiality [36], and the effect of ecological interactions between coexisting lineages on the effects of mutations [37]. None of these LTEE-based studies documented a reduction in the mean of the distribution of fitness effects. However, Limdi et al. [36] showed that more genes transitioned from non-essential to essential than vice versa. That is, insertions in some genes became highly deleterious or lethal over the course of evolution.

Together, these studies suggest that as microbial populations adapt to laboratory conditions, deleterious insertion mutations show a tendency towards becoming more deleterious (increasing-costs), though their effects change less consistently than beneficial mutations. Again, the key question is when and why we expect to see increasing-costs epistasis leading to a reduction in mutational robustness. How do these phenomena depend on the environment, biological system, or evolutionary timescale?

## Patterns of epistasis within proteins

To start asking whether we should expect to see patterns of global epistasis in other systems or on other timescales, we first consider another level of biological organization on which data on epistasis is being rapidly accumulated: within-protein evolution. The advent of deep mutational scanning technologies (which screen the local mutational neighborhood around a focal genotype; reviewed in [45] and [46]), along with ancestral reconstruction techniques and high-throughput assays for protein function, have opened the door to directly study how the array of mutational effects possible for a given protein sequence changes as the protein evolves (Fig. 2).

Deep mutational scanning studies have frequently observed a pattern of global epistasis in which mutations have additive effects on an unobserved trait (which presumably represents one or several biophysical properties such as stability or enzyme activity) that in turn maps nonlinearly to the observed phenotype (often binding affinity or fluorescence) [15, 47]. This simplification of the genotype–phenotype map allows us to predict the effects of mutations using relatively few parameters (i.e., the additive effects of mutations on the unobserved trait and the form of the nonlinearity). However, we note that this form of global epistasis is distinct from fitness-correlated global epistasis: for example, two mutations that have the same effect on one genetic background will have the same effect on another, which is not necessarily the case for fitness-correlated epistasis.

### Epistatic drift makes the effects of mutations less predictable as proteins evolve

While the models of within-protein global epistasis discussed above were built from deep mutational scanning datasets in which genotypes differ by a few mutations, these methods have now been combined with ancestral sequence reconstruction to study epistasis along long-term evolutionary trajectories. For example, recent work has quantified how the effects of mutations change as the DNA binding domain of steroid hormone receptors evolved over 700 million years [48], and as the receptor-binding domain (RBD) of the spike protein evolved in the family of sarbecoviruses containing SARS-CoV-2 [4]. It is difficult to look for phenotype-correlated epistasis (e.g., diminishing-returns) in these datasets for three reasons: (1) there are fewer genetic backgrounds than in multi-mutation deep mutational scanning datasets, (2) in some cases (e.g., Park et al. [48]), we do not have measurements of the phenotypes of the relevant genetic backgrounds, and (3) the limited dynamic range of these assays can present statistical challenges. Presumably for these reasons, neither of these studies specifically investigate phenotype-correlated epistasis. However, as these approaches grow more common and produce even larger datasets, it will be interesting to study whether patterns of global epistasis hold predictive power over long evolutionary timescales.

While these studies do not directly analyze global epistasis, they do find a consistent pattern: the effects of different mutations “drift” randomly during evolution, and each mutation has a different characteristic speed at which its effect drifts. Park et al. [48] show that these mutation-specific rates of change are consistent in different parts of the phylogenetic tree (proportional to the number of background substitutions on the branch). Starr et al. [4] find a similar pattern, in which mutational effects at different sites in the RBD drift across increasingly different genetic backgrounds, again with site-specific speeds. Together, these studies show that certain mutations are more likely to have unchanging effects over evolutionary timescales, while others will change rapidly as a result of epistatic patterns that are currently difficult to simplify. These different rates of epistatic drift, which can in principle be estimated from much smaller datasets, can tell us how likely it is that the phenotypic effect of mutating a particular site is similar to its effect on another background. This is a useful parameter for large-scale Bayesian models seeking to predict epistasis [49].

## Models of global epistasis

Our best hope for building predictive models of epistasis during evolution is to reduce the dimensionality of the mapping between genotypic configurations and fitness, often referred to as the fitness landscape. The commonality of global or fitness-correlated epistasis suggests that these reductions are *sometimes* possible, but as we have seen above, these patterns are not universal. Instead, they depend on environmental conditions, the level of biological organization considered, and the genetic distance over which we are trying to predict epistasis. What we need, then, are models that can tell us, using either biological knowledge and/or sparse datasets as inputs, what forms of epistasis to expect. In this section, we will review a few nascent efforts towards this end.

### Statistical models

One way to understand the emergence of patterns such as fitness-correlated epistasis is to describe statistical models of the genotype to phenotype map that could lead to such phenomena (Diaz-Colunga et al. [50] provide an in-depth review of these types of explanations for patterns of global epistasis). Recent studies [51, 52] have proposed models where diminishing-returns and increasing-costs epistasis arise when a mutation has a large number of epistatic interactions with the genetic loci that vary in the background. These studies show that extensive idiosyncratic epistasis can lead to a general pattern of fitness-correlated global epistasis. Intuitively, in a highly epistatic fitness landscape, fitter genotypes will tend to have a larger excess of positive (relative to negative) interactions between loci. Consequently, a mutation that interacts with many of these loci will disrupt, on average, a larger number of positively-contributing interactions when it occurs on a fitter background. Provided that the mutation has numerous interactions with the background loci, a mathematical argument based on the central limit theorem shows that the mutation’s fitness effect has a negative linear dependence on background fitness, irrespective of whether the mutation is beneficial or deleterious on average [51, 52].

**Fig. 3:**
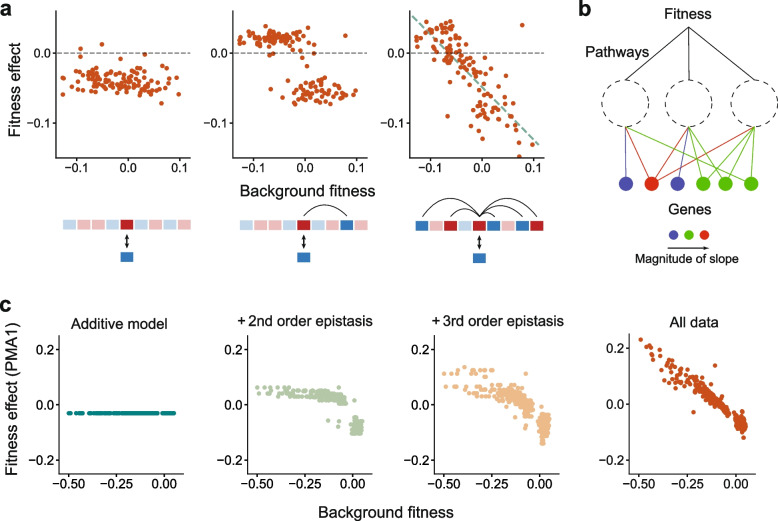
Statistical models of epistasis. **a** The fitness effects of three example insertion mutations on background strains obtained from a yeast cross (data from [44]). These examples illustrate a mutation that shows no detectable epistatic interactions (left), a mutation that has a specific interaction with one locus in the background (middle) and a mutation that shows a negative linear trend (right), which arises if a mutation has many idiosyncratic interactions with background loci. **b** Theory predicts that the slope of the linear global epistasis trend depends on the number and magnitude of interactions the mutation has with background loci. The variability in slopes across mutations can potentially be explained by their involvement in a variable number of pathways that contribute to fitness. **c** An empirical fitness landscape with ten mutations (85% complete) shows a linear global epistasis trend for mutation PMA1. This trend emerges as terms of increasing orders of epistasis are added to the model. Data from [53]

In this framework, mutations can be broadly classified into three classes: (1) additive mutations that have an effect independent of the background, (2) mutations which have strong and specific epistatic interactions with one or a few other genetic loci, and whose fitness effect can be positively or negatively correlated with background fitness, and (3) mutations that have epistatic interactions with numerous loci and thereby show a negative linear dependence on background fitness. Figure 3 shows example mutations from [44] that correspond to these three cases. The model predicts that for mutations of the third class, the *negative slope* of the linear trend is proportional to the *variance of the residuals* around this trend, which is consistent with data from Bakerlee et al. [53].

Another feature of the data from Bakerlee et al. [53] is that the negative slope of the fitness-correlated trend varies significantly across mutations. This variation in slope could arise if mutations with steeper slopes are involved in more biological pathways that contribute to fitness, and therefore have a larger number of interaction terms (Fig. 3b). [52] introduced a Gaussian fitness landscape model to quantify this intuition, which also predicts that the rate of adaptation should decline as a power law with time and that the DFE of beneficial mutations should be exponential when the organism is well-adapted to the environment. If acquired mutations are uncorrelated with fitness, this model also leads to epistatic drift at a site-dependent rate.

While this theory assumes that mutations have numerous epistatic interactions with other loci, diminishing-returns epistasis has also been observed across lineages that have acquired fewer than ten mutations. For example, a recent study used a hierarchical CRISPR gene drive system to construct 875 of the 1024 possible combinations of 10 mutations that affect diverse functions in yeast [53]. By directly measuring higher-order epistasis terms, this work shows that fitness-correlated linear trends can emerge even from a small number (~ 5–10) of idiosyncratic interactions (Fig. 3c).

### Functional models

Another class of explanations for patterns of global epistasis derives from a mechanistic perspective on biological systems. Lehner [54] provides an excellent review of a diverse set of potential molecular mechanisms underlying epistasis, including protein stability thresholds, functional redundancy, and metabolic pathway structure. Here we review recent work that has attempted to devise functional models of global epistasis.

**Fig. 4:**
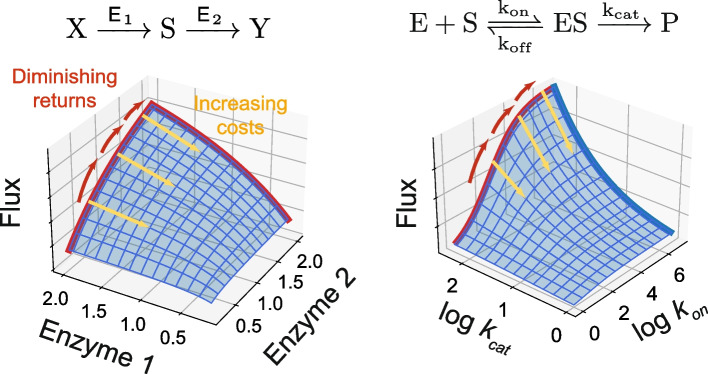
Diminishing-returns and increasing-costs epistasis in simple models of enzyme kinetics. (Left) The dependence of flux on two enzymes in a serial metabolic pathway, given by $J = a/(1/{E}_{1 }+ 1/{E}_{2} + b)$ [56]. (Right) Michaelis–Menten kinetics. In the unsaturated regime, the downstream flux depends multiplicatively on ${k}_{on}$ and ${k}_{cat}$. Mutations that increase $\log {k}_{\text{on}}$ experience diminishing-returns, whereas mutations that reduce $\log {k}_{\text{cat}}$ incur larger costs at larger values of $\log {k}_{\text{on}}.$

Several studies have attempted to explain patterns of fitness-correlated epistasis through the lens of metabolic control theory (MCT), which is a framework used to relate the sensitivity of the flux through a metabolic pathway to changes in enzyme concentration or to mutations that modulate enzyme kinetics [55, 56]. MacLean [57] applied the basic ideas of MCT beyond metabolic pathways into the core processes of transcription and translation, showing that strains that grow more slowly are more robust to a ribosome-inhibiting antibiotic. This relationship provides a functional explanation for increasing-costs epistasis in transcription or translation machinery: as flux through this pathway increases, mutations become more costly. We have also recently used an MCT framework to rationalize why patterns of diminishing-returns and increasing-costs arise in microbial evolution experiments [11]. We explore these ideas further in Box 2 and Fig. 4.

More recently, Kryazhimskiy [58] used MCT to show how epistasis between two mutations within a given sub-pathway can change as we zoom out to consider their effect on the larger pathways in which this sub-pathway is embedded. Under a linearity assumption, this analysis finds a non-trivial asymmetry in how positive and negative epistasis propagate: negative epistasis at smaller scales remains negative on larger scales, while positive epistasis can change sign. This could lead to a skew in the distribution of epistatic coefficients that can in principle lead to global epistasis, though the paper is careful to point out that the specific structure of the metabolic network itself can also lead to different likelihoods of negative and positive epistasis.

Husain and Murugan [59] take an alternative physics-inspired approach, relating patterns of global epistasis in proteins to the collective relaxation modes of protein conformations. For example, if a single mode dominates the relaxation dynamics of a protein and the measured phenotype is predominantly influenced by protein conformation, then mutational effects will show changes primarily along this dominant relaxation mode. If these mutational effects act additively on the dominant mode, the genotype–phenotype map can be approximated as a nonlinear function applied to an additive trait. More generally, if the relaxation dynamics of a system are dominated by $k$ modes, then epistatic effects should span a $k$-dimensional subspace, provided that mutational effects are small and specific epistasis mediated by direct contacts is negligible. This argument provides a physical explanation for global epistasis, which can be further tested by measuring the dimensionality of epistatic effects (possibly acquired through deep mutational scans) on a protein and its normal mode spectrum.

## Predicting epistasis, predicting evolution

### Inference of landscape structure

While many of the models described in the previous section make a priori predictions about global epistasis, we can also use them alongside data-driven inference approaches that aim to find simpler patterns in epistatic effects. A common goal of these methods is to predict the phenotypes of unobserved genotypes using a relatively limited set of genotype–phenotype measurements.

One conceptually straightforward approach to this problem is to model the fitness of each genotype as a sum of additive effects of individual mutations, along with pairwise and higher-order interactions between them. We can then infer the additive effects and interaction coefficients (typically up to some maximum order, and potentially with some regularization term) from genotype–phenotype data. This defines a model that can be used to predict phenotypes of unobserved genotypes. Bakerlee et al. [53] and Poelwijk et al. [60] took this approach to infer epistatic effects in combinatorially complete or near-complete libraries spanning sets of mutations across and within genes, respectively. Both found that a relatively small number of epistatic terms can explain the vast majority of the variation in phenotype. In other words, strong epistatic effects on phenotypes are sparse, such that it is possible to accurately predict phenotypes for the entire set based on a sparse sample of phenotypic measurements (e.g., 6–11% of the combinatorially complete set of measurements in Poelwijk et al. [60]). Poelwijk et al. [60] also demonstrates that epistatic coefficients can be accurately inferred from the subset of sequences that cross some phenotypic threshold, suggesting that it may be possible to predict these coefficients from the set of extant, presumably functional, sequences of a protein.

A second category of inference uses genotype–phenotype data to infer the functional architecture of biological systems, which can later be used to predict the effects of mutations. The most comprehensive work in this direction has aimed to characterize the genetic organization of budding yeast based on measurements of epistatic interactions between gene knockouts and knockdowns [1]. While this type of work can provide the foundation for genome-scale models of microorganisms that predict how changes in gene activities will affect phenotypes (reviewed in Fang et al. [61]), the data generally requires careful curation and typically cannot model the effects of individual mutations.

A third category of approaches is to assume that the genotype–phenotype map has a low-dimensional structure. For example, a global epistasis model analyzed by Otwinowski et al. [47] assumes that the observed phenotype is a nonlinear function of a continuous additive trait. To build a predictive model, these authors use training data to infer the effect of each mutation on the additive trait, as well as the nonlinear function that maps this trait to the measured phenotype. An alternative approach is to use some dimensionality reduction method on large-scale genotype–phenotype data sets to infer the map between genotype and a low-dimensional latent space and between the latent space and observed phenotypes. For example, [62] used singular value decomposition to infer a latent space, and then used the inferred structure to predict the fitness effects of many mutations across many environments in yeast.

These dimensionality reduction approaches are also present in new machine-learning methods for predicting patterns of epistasis, which we expect to become more common and accurate in the coming few years [63, 64]. For example, Tareen et al. [64] present a flexible method for learning the relationship between genotype and phenotype. This method includes additive, pairwise, or neural-net-based models to produce a single latent phenotype, which may then be related to the measured phenotype through a nonlinear function, as described in Otwinowski et al. [47]. In contrast, Tonner et al. [63] use a hierarchical Bayesian model that explicitly attempts to identify a relatively small number of uncorrelated latent variables that collectively determine phenotypes. In three within-protein datasets, their model identifies 3–5 latent dimensions, some of which may be interpretable in terms of biologically meaningful traits such as ligand binding affinity.

Given good data, the goal of predicting fitness effects of mutations is well-defined, in the same way that protein structure prediction has been well-defined for decades. A combination of machine learning and functionally motivated approaches should make high-accuracy phenotype prediction possible, at least among relatively closely related genotypes. The question will be whether we can predict effects more widely using sparse or mutationally distant datasets. Currently, we are limited both by data and by our modeling approaches. As these both improve, we will approach the limits set by the true dimensionality of the phenotypes produced by biological systems.

The concept of “epistatic drift” provides one simple framework to think about these limitations: we should expect our ability to predict epistasis to decline as the genetic distance between the backgrounds in our data set and our prediction target increases, and we should expect this effect to vary between mutations [4, 48]. Importantly, we can use this relationship between genetic distance and fitness correlations to build priors that take into account the structure of higher-order epistasis [49]. As we gather more high-throughput datasets on within-protein epistasis, we hope it will be possible to connect these patterns of epistatic drift to functional and structural properties of proteins, such that even when using sparse mutation data, structural information can be used to bolster predictive models.

### Predicting evolution

Characterizing how the fitness effects of mutations change during evolution is essential if we are to predict evolutionary dynamics. The work reviewed here shows that this is a difficult task. While the effects of some mutations follow predictable fitness-correlated patterns, these patterns are not universal: they depend on the biological system and phenotype under selection (e.g., a protein’s binding efficiency, a microbial cell’s growth rate) and the timescale (e.g., strains separated by few mutations and generations or by hundreds of thousands of mutations and generations). We are cautiously optimistic that as we gain the ability to predict epistasis in different systems and to identify the functional basis of the latent phenotypes that generate this epistasis, we will improve our ability to predict when phenomena such as declining adaptability or robustness, among other patterns of changing mutational effects, will occur as cells or proteins evolve in nature.

Box 1: Epistasis terminology Epistasis is a concept laden with overlapping terminology. Here we provide a brief glossary and comparison of terms.

*Epistasis*: When the effect of a mutation on a phenotype, such as fitness, depends on the genetic background on which it arises. For two mutations, epistasis is quantitatively defined as the sum of effects of the individual mutations subtracted from the effect of the double mutant.

*Positive versus negative epistasis*: Epistasis between two mutations is positive (negative) when the effect of the double mutant is greater (smaller) than the sum of the effects of the two individual mutations.

*Synergistic versus antagonistic epistasis*: Epistasis between two beneficial or two deleterious mutations is synergistic (antagonistic) if the absolute value of the effect of the double-mutant is larger (smaller) than the absolute value of the sum of the effects of the two individual mutations. Note that this is subtly different from the distinction between positive and negative epistasis.

*Sign/magnitude epistasis*: When the mutation’s effect across genetic backgrounds varies in magnitude but not in sign (magnitude epistasis) or when its sign also varies (sign epistasis).

*Statistical epistasis*: The *average* epistasis between two mutations when measured on genetic backgrounds (at other loci) that are randomly sampled from a population.

*Distribution of Fitness Effects (DFE)*: The probability that a random new mutation has a particular fitness effect. The DFE can in principle vary depending on the genetic background on which the mutations arise.

*Macroscopic versus microscopic epistasis*: The dependence of the distribution of fitness effects on the genetic background (macroscopic epistasis), in contrast with the dependence of effects of specific mutations on the genetic background (microscopic epistasis).

*Specific/idiosyncratic epistasis*: When the fitness effect of a mutation depends in some specific way on the allelic state at other sites, for example, due to protein–protein interactions or direct contact between residues in a protein, we refer to this as “idiosyncratic” epistasis because it depends on all the particular details of these physical interactions.

*Global/unidimensional/nonspecific epistasis*: When the effect of a mutation on diverse genetic backgrounds can be described using relatively few parameters. In microbes, global epistasis often manifests as a linear dependence of the mutation’s fitness effect on background fitness. In proteins, global epistasis generally refers to a situation where the measured phenotype is well-approximated by a nonlinear function of an unobserved additive trait.

*Diminishing-returns and increasing-costs epistasis*: Forms of global epistasis where the fitness effect of a mutation is negatively correlated (often linearly) with background fitness. When the mutation is on average beneficial this is referred to as diminishing-returns, while if it is deleterious it is termed increasing-costs (a mutation can exhibit both if its effect crosses zero as a function of background fitness).

*Functional epistasis*: Distinct from the genetic definition of epistasis, this term is used to describe protein–protein interactions, for example between proteins that physically interact or belong to the same pathway.

Box 2: A speculative model of diminishing returns and increasing costs in evolving microbial populations Why do we sometimes observe fitness-correlated epistasis in evolving microbial populations? Let’s speculate!

First, let’s make a distinction about redundancy, by thinking about two extreme functions which describe how biological phenotypes (e.g., the activities of genes, pathways, or modules) are integrated to produce a higher-level phenotype.

**Function 1: Maximum.** The two phenotypes are completely redundant, such that the higher-level phenotype depends only on the maximum value of the two lower-level phenotypes.

**Function 2: Product.** The higher-level phenotype is the product of the lower-level phenotypes, such that they are non-redundant and the limiting lower-level phenotype primarily controls the higher-level phenotype.

These two cases qualitatively correspond to parallel and serial pathways, respectively.

Our first claim here is that while Function 1 is likely to underlie many specific cases of epistasis (e.g. synthetic lethals), Function 2 is, on the whole, more common in microbial cells under selection for growth, because the core processes of growth and the processes underlying growth largely work with non-redundant components.

Our second claim is that selection in laboratory microbial evolution experiments is primarily for *loss-of-function *mutations clustered in a small number of biological modules. Consistent with this hypothesis, numerous experiments have found that replicate populations fix loss-of-function mutations in similar pathways, even though the specific mutations differ across replicate lines. Notable examples in yeast include repair mutations that compensate for disrupted pathways (e.g the adenine biosynthesis pathway) and mutations in nutrient response pathways (e.g., Ras/PKA, TOR/Sch9), which integrate intracellular and extracellular signals and act as key regulators of gene expression. Within these modules (and perhaps between them), beneficial loss-of-function mutations will interact negatively: because the genes act non-redundantly to produce a function that is detrimental to fitness, mutations breaking them will exhibit diminishing-returns epistasis.

Since we have claimed that Function 2 is more common, we can think of the effect this has on cellular fitness in a simple multiplicative model:

$F={\prod\nolimits_i}\alpha_i$, where $\alpha_i$ represents the efficiency of some process related to fitness.

As populations fix beneficial loss-of-function mutations and increase the value associated with the module under selection, the cost of decreasing any of the unchanged values will become more severe (increasing costs).

Notably, this model implies that when we see *gain-of-function* beneficial mutations in core, non-redundant processes related to growth, we expect to see positive epistasis between them. Chou et al. [40] provides a striking example of this pattern, in which a promoter capture mutation which increases cobalt uptake is more beneficial in faster growing strains. In this context, it seems likely that the pattern of declining adaptability will often arise during short-term adaptation to a new environment, but might not over longer evolutionary timescales or when adaptation proceeds primarily through gain of function (e.g., as the result of horizontal gene transfer)

## Data Availability

Not applicable.

## Abbreviations

DFE: Distribution of fitness effects
LTEE: Long-term evolution experiment
RBD: Receptor binding domain
MCT: Metabolic control theory
